# The ME-BYO index: A development and validation project of a novel comprehensive health index

**DOI:** 10.3389/fpubh.2023.1142281

**Published:** 2023-05-05

**Authors:** Sho Nakamura, Ryo Watanabe, Yoshinobu Saito, Kaname Watanabe, Ung-il Chung, Hiroto Narimatsu

**Affiliations:** ^1^Graduate School of Health Innovation, Kanagawa University of Human Services, Kawasaki, Japan; ^2^Cancer Prevention and Control Division, Kanagawa Cancer Center Research Institute, Yokohama, Japan; ^3^Center for Innovation Policy, Kanagawa University of Human Services, Kawasaki, Japan; ^4^Faculty of Sport Management, Nippon Sport Science University, Yokohama, Japan; ^5^Department of Genetic Medicine, Kanagawa Cancer Center, Yokohama, Japan; ^6^Department of Bioengineering, Graduate School of Engineering, The University of Tokyo, Tokyo, Japan

**Keywords:** healthy aging, intrinsic capacity, quality of life, activities of daily living, smartphone, non-communicable disease

## Abstract

Quantifying health status and identifying modifiable factors are essential for effective and individualized prevention of age-related conditions and for promoting health during aging. The ME-BYO concept from Kanagawa Prefecture, one of Japan’s largest prefectures, can be used to establish a healthy aging society. In disease etiology, ME-BYO considers the status of an individual’s body and mind as changing continuously from healthy to sick instead of being a dichotomy between the two. ME-BYO conceptualizes the entire process of this change. The ME-BYO index was developed in 2019 to comprehensively and numerically measure and visualize an individual’s current health status and future disease risk by quantifying data on the four domains of metabolic function, locomotor function, cognitive function, and mental resilience. The ME-BYO index has been implemented in the personal health management application “My ME-BYO.” However, scientific validation of this index and the development of a practical application using healthcare data remain to be completed. In 2020, our research team started a project to refine the ME-BYO index using data from the Kanagawa ME-BYO prospective cohort study, which is a large population-based genomic cohort study. This project will scientifically evaluate the ME-BYO index and develop a practical application for promoting healthy aging.

## 1. Introduction

Non-communicable diseases (NCDs) tend to be caused by a combination of genetic, physiological, environmental, and behavioral factors and are among the most concerning global issues in healthcare (1). According to the World Health Organization (WHO), NCDs accounted for 71% of global deaths in 2015 (2). The WHO has promoted a “Healthy Aging” policy focused on maintaining an individual’s functional ability, which combines their intrinsic capacity and environmental characteristics (3). The prevention of NCDs may reduce the risk of intrinsic capacity decline. As one of the most-aged countries, Japan has worked to prevent the spread of NCDs. In 2000, the Ministry of Health and Welfare introduced “Health Japan 21,” a national health promotion policy aimed at prolonging healthy life expectancy by preventing NCDs through lifestyle improvement (4). The following six categories of detailed target values and prevention strategies are set in Health Japan 21: nutrition and dietary habits, physical activity and exercise, rest, alcohol drinking, tobacco smoking, and oral health (5).

Multiple factors, including lifestyle, determine an individual’s health status. Therefore, both quantifying a person’s health status and identifying modifiable factors are essential for effectively and individually preventing age-related conditions and promoting health during aging. Kanagawa Prefecture, one of Japan’s largest prefectures, is pursuing the “Healthcare New Frontier” policy initiative, which aims to establish a society where everyone can live a long and healthy life (6). In the Kanagawa Prefecture ME-BYO concept of disease etiology, the body and mind states are considered to shift continuously between healthy and sick instead of being a dichotomy between the two. ME-BYO measures and visualizes the entire change process and can incentivize a patient to change their behavior. ME-BYO can be translated as “no disease” and viewed as a dynamic stage of transitioning from being healthy to the onset of full-blown disease and disability. At this stage, preventive measures may effectively maintain or reverse the disease trajectory. The ME-BYO index was developed in 2019 to comprehensively and numerically measure an individual’s current health status and future disease risk by quantifying data on the four domains of metabolic function, locomotor function, cognitive function, and mental resilience. The ME-BYO index is implemented in the personal health management application “My ME-BYO personal health record” (7).

The ME-BYO index was developed based on expert discussions. However, to promote changes in individual behavior effectively, it is necessary to refine the index further by analyzing high-quality data from well-established cohorts of older adults, evaluating the predictive value of the index, and examining associations between health behavior and lifestyles. In addition, the index should be scientifically validated, and its practical applications require development. In 2020, our research team started a project to refine the ME-BYO index. Since then, we have promoted behavioral changes in the prefecture residents by conducting demonstrations and refined the ME-BYO index to add predictive functions based on analysis of the Kanagawa ME-BYO Prospective Cohort Study (8), a population-based genome cohort study. A prospective cohort study is the only study design to achieve this refinement of ME-BYO because it can collect data on the process of healthy people contracting some diseases.

In this study, we review the concept of ME-BYO, present a study design for refining the ME-BYO index, and discuss the use of this index for establishing a healthy aging society (3).

## 2. The ME-BYO index

### 2.1. The ME-BYO concept

The “Healthcare New Frontier Policy” of Kanagawa Prefecture aims to create a society where everyone can live healthily in a super-aged society (6). This policy comprises mainly the following two approaches: (1) realizing personalized health through facilitating cutting-edge medicine and technology and (2) reviewing personal lifestyles through improving individuals’ ME-BYO. The ME-BYO concept focuses on integrated health status, which resists a future decline in intrinsic capacity.

### 2.2. Development of the ME-BYO index

Healthy behavior and lifestyle are essential for improving ME-BYO status when people are at risk of developing an NCD; however, encouraging people to change their behavior requires time and effort. The Health Belief Model evaluates health behavior using the following six key concepts: perceived susceptibility, perceived severity, perceived benefits, perceived barriers, cue to action, and self-efficacy (9). Based on this model, it is necessary to perceive the risk accurately and understand the benefits of behavior change to induce ME-BYO-improving behavior. The ME-BYO index was developed in 2019 to promote behavioral changes by visualizing an individual’s ME-BYO status. The ME-BYO index was designed to assist users in understanding their current and projected ME-BYO states.

The ME-BYO index is scored based on a 15-item measure across the following four domains: metabolic function, locomotor function, cognitive function, and mental resilience. The WHO categorizes intrinsic capacity into the following five parts: physical mobility, vitality, psychosocial, sensory, and cognitive (10). The ME-BYO index was designed using this approach; it is calculated by summing the weighted partial scores that assess the status of the four domains. Various scientifically-based indicators were collected using a survey to build a scoring formula for domain-specific assessments. The following measurement indices were used to calculate the ME-BYO index (Table 1): the three-question version of the Mini-Cog (11–13) was used to evaluate cognitive function; the five-question Geriatric Locomotive Function Scale questionnaire (14) and walking speed measured by smartphone, were used to assess living function; and individual voice information was used to evaluate mental health and stress. The Mind Monitoring System (MIMOSYS; PST Inc., Yokohama, Japan) has been recently developed (15–17) and implemented to evaluate mental resilience using smartphone applications.

**Table 1:** Current version of the ME-BYO index.

Domains	Items
Metabolic function	Sex, age, body mass index, systolic blood pressure
Cognitive function	Mini-Cog (3 questions)
Locomotor function	locomotive syndrome questionnaire (Locomo 5; 5 questions), walking speed
Mental resilience	MIMOSYS (voice)

MIMOSYS, the Mind Monitoring System.

## 3. Design of the Kanagawa ME-BYO prospective cohort study

### 3.1. Background of the Kanagawa ME-BYO prospective cohort study

Research on gene–environment interactions has led to the development of personalized preventive medicine that considers both environmental and genetic factors and the characteristics of each patient (18, 19). One of the aims of the Kanagawa ME-BYO prospective cohort study (ME-BYO cohort) is to clarify gene–environmental interactions in NCDs in collaboration with the J-MICC Study (20). Details of the ME-BYO cohort and the J-MICC Study and the relationship between the two have been described in detail elsewhere (20, 21). The ME-BYO cohort was designed to perform original research on disease-prevention methods that are more efficacious than conventional measures to contribute to Kanagawa Prefecture’s healthy-aging policy based on the ME-BYO concept. The original ME-BYO cohort study aimed to evaluate the risks of developing diseases, which can damage an individual’s quality of life (QOL), include genetic factors related to NCDs, and use data from the ME-BYO cohort to develop an effective strategy for healthy aging based on the ME-BYO concept.

### 3.2. Study design

People aged 18–95 years who are residents of, or are working in, Kanagawa Prefecture were recruited as participants in the ME-BYO cohort; baseline recruitment and surveying began in 2016 and will continue until 2023. Participants were recruited during health-checkup run by municipalities or companies. We recruited some participants to the baseline survey directory. In addition, the baseline survey included a self-administered questionnaire and health examinations of the ME-BYO cohort. The self-administered questionnaire collected data on sociodemographic characteristics, including sleeping, physical activity, alcohol consumption, smoking, psychological stress, use of medications and supplements, diet, personal and family disease history, personality and psychological state, social relationships, relationship to mass media, mobility, QOL, defecation, and female reproductive history. Data were also collected from physical check-ups and laboratory findings, including blood chemistry and complete blood cell count data. For genomic analysis and bio-banking, blood was drawn into plain tubes for serum assays and ethylenediamine tetraacetic acid-containing tubes for plasma and buffy coat assays. Urine samples and stool samples were collected from some of the participants.

The target sample size was 5,000 people. The ME-BYO cohort was originally started to investigate gene-environmental interaction. The sample-size requirements in prospective cohort studies were already calculated (19). It indicated the large sample size needed. We will collaborate with J-MICC or other large cohort studies in Japan to conduct such investigations requiring a large sample size.

Follow-up data is planned to be collected through the population-based Kanagawa Cancer Registry (22, 23), where questionnaires are mailed to the participants, questionnaires are administered at follow-up visits to health check-up facilities, and information from death certificates and health insurance data will be collected. The endpoints of the follow-up study will be incident disease diagnoses, which include cancer, cardiovascular disease, stroke, and death from any cause. Biological phenotypes and biomarkers, including laboratory test findings, were also used.

## 4. Development and validation of the ME-BYO index

### 4.1. Retrospective analysis using the ME-BYO cohort

**Figure 1:**
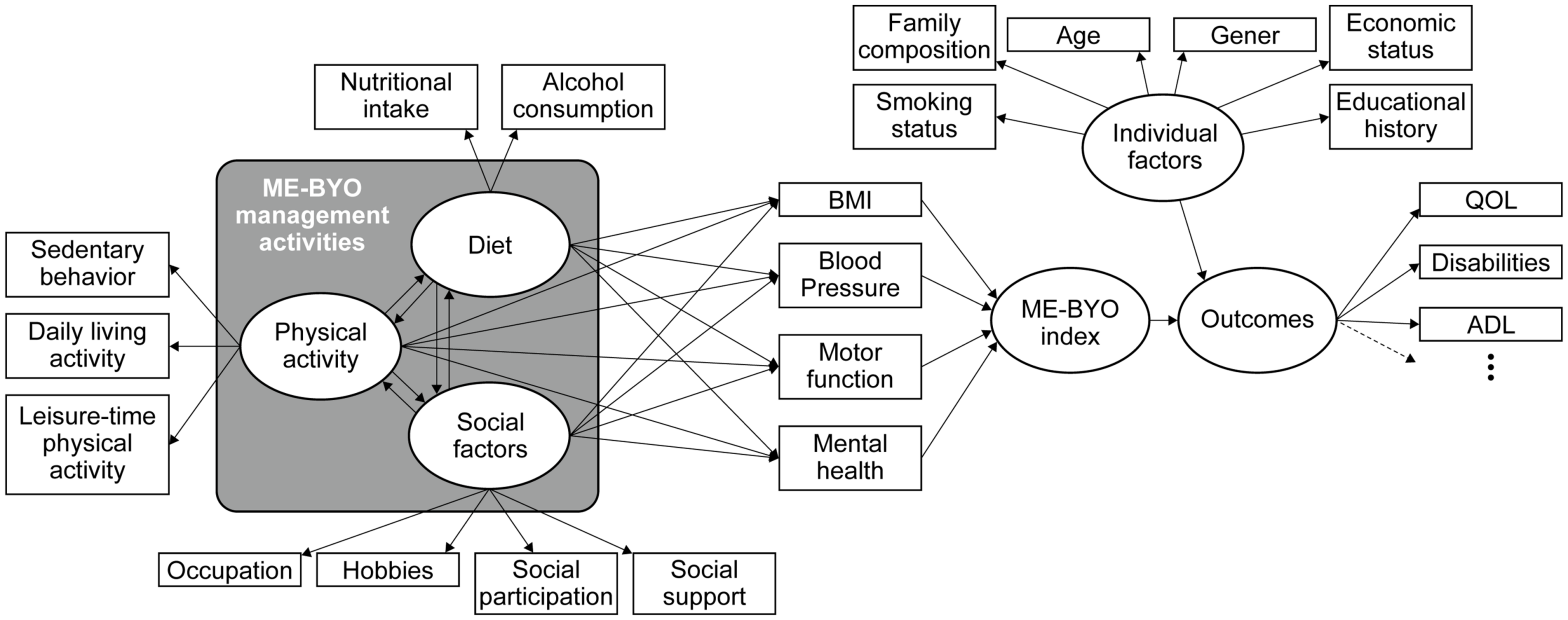
An example of a structural equation model. Domains of the ME-BYO index using explained variables and structural equation models with quality of life (QOL)-related indicators as outcome variables are shown. BMI, body mass index.

We are planning to analyze data from the ME-BYO cohort using structural equation modeling analysis as a previous study about intrinsic capacity used (24). The main aim of the analysis is to verify the concept and existence of the ME-BYO index. Our hypothetical model is shown in Figure 1. Briefly, the ME-BYO index is treated as a latent variable in the analysis, which comprises observed variables that are the domains of the current scoring method. Cognitive function is excluded since it is not collected in the ME-BYO cohort. MIMOSYS is not collected either; therefore, Kessler’s Psychological Scale (K6) (25) was used as a substitute for the MIMOSYS. We hypothesized that four domains are affected by the ME-BYO management activities, and we also assumed that the ME-BYO management activities, diet, physical activity, and social factors affect each other. We will select the outcomes, shown as a latent variable in Figure 1, to estimate the ME-BYO status that also reflects the intrinsic capacity. For example, for QOL-related measures, such as the EQ-5D-5L (26, 27) and one-item subjective happiness, the participants were asked, “How happy do you feel about your life?” (28) and about laughter frequency. Data that are to be collected in the follow-up survey are planned to be used in the analysis.

### 4.2. Prospective analysis using the ME-BYO cohort

A validation study performing the Mini-Cog and walking test on a smartphone has been conducted (11, 29); these comprise the ME-BYO index. The ME-BYO index, evaluated on smartphones or tablets, has been included in the baseline survey since 2020. A follow-up survey was performed to obtain data on the long-term QOL outcomes, activities of daily living, instrumental activities of daily living, healthy life expectancy, medical costs, and nursing care costs. These are the endpoints of the ME-BYO index. A 1-year follow-up survey of baseline survey participants will examine the relationship between changes in the ME-BYO index and activities that improve wellness (diet, exercise, and social participation) and QOL. Subsequently, we will consider implementing a more precise MY-BYO index than that has already been adopted in the smartphone application. Some additional analysis that would be important in healthy aging can be conducted in the future, including the impact of education programs using the ME-BYO index on healthy lifestyles and emotional health and the financial and social impact of investing in improvements in the quality of an aging population, such as the “Healthcare New Frontier Policy” of Kanagawa Prefecture. In all those analyses, a *p* < 0.05 is considered statistically significant.

## 5. Discussions and future directions

This article presents the concept of the ME-BYO index and its validation using a genomic-cohort study. This project will clarify the scientific validity of the ME-BYO index and its significance in establishing healthy aging. However, several issues remain to be addressed. First, the endpoint of the index should be defined clearly. The ultimate endpoint is to show that a higher score on the ME-BYO index is associated with healthy longevity; this requires a follow-up period of at least 10 years. Although EuroQol 5 Dimension and subjective happiness could be used as alternative endpoints, they might not necessarily be a good substitute for this ultimate goal. The project plans to obtain future data on nursing care and medical costs, which will enable us to analyze this relationship. However, the optimal endpoint may differ among subgroups; for example, it may naturally differ between generations or between males and females. Therefore, an exploratory analysis using cohort studies is required to evaluate these potential differences.

The weighting strategy for each domain should be re-evaluated. The first version of the ME-BYO index was calculated using weights based on published studies of healthy aging; however, new data-based weights can be derived from the actual data of the ME-BYO cohort. The individualization or stratification of the weighting strategy should also be investigated. The ME-BYO index’s clinical or public health significance will probably vary depending on sex, and the purposes for which the ME-BYO index is used may differ significantly. Notably, excessive individualization may reduce the advantage of the indicator, which is comprehensive and universal to everyone, and the optimal approach may vary depending on the intended use.

A practical application for healthy aging should be developed for the ME-BYO index. The most significant advantage of this index is its ability to measure an individual’s health status. Measuring the effects of preventive medical interventions, such as exercise and diet therapy, is a promising method for using the index compared to a conventional health check comprising a blood test and physical examination by a medical doctor. Therefore, we plan to evaluate the significance of various preventive medical interventions in the future.

## Author contributions

SN, RW, and HN wrote the manuscript. SN, RW, YS, KW, HN, and U-iC contributed to the study conception and design. All authors contributed to the article and approved the submitted version.

## Funding

Kanagawa Prefectural Government, Japan, supported this ME-BYO index development project. The ME-BYO cohort was in part supported by the Japan Society for the Promotion of Science (JSPS) KAKENHI Grant (no. 16H06277 (CoBiA)) from the Japanese Ministry of Education, Culture, Sports, Science and Technology.

## Conflict of interest

The authors declare that the research was conducted in the absence of any commercial or financial relationships that could be construed as a potential conflict of interest.

## Publisher’s note

All claims expressed in this article are solely those of the authors and do not necessarily represent those of their affiliated organizations, or those of the publisher, the editors and the reviewers. Any product that may be evaluated in this article, or claim that may be made by its manufacturer, is not guaranteed or endorsed by the publisher.

## References

[1] Benziger CP Roth GA Moran AE. The global burden of disease study and the preventable burden of Ncd. Glob Heart. (2016) 11:393–7. doi: 10.1016/j.gheart.2016.10.024 27938824

[2] WHO. Non-communicable diseases [home page on the internet]. (2022). Available at: https://www.who.int/news-room/fact-sheets/detail/noncommunicable-diseases. (updated September 16; Accessed December 26, 2022).

[3] Rudnicka E Napierala P Podfigurna A Meczekalski B Smolarczyk R Grymowicz M. The World Health Organization (WHO) approach to healthy ageing. Maturitas. (2020) 139:6–11. doi: 10.1016/j.maturitas.2020.05.018, PMID: 32747042 PMC7250103

[4] Nomura S Sakamoto H Ghaznavi C Inoue M. Toward a third term of health Japan 21 – implications from the rise in non-communicable disease burden and highly preventable risk factors. Lancet Reg Health West Pac. (2022) 21:100377. doi: 10.1016/j.lanwpc.2021.100377, PMID: 35098183 PMC8783949

[5] Health Japan 21 (The Second Term) Analysis and Assessment Project [Home page on the internet] (2013). Available at: https://www.nibiohn.go.jp/eiken/kenkounippon21/en/kenkounippon21/. (Accessed December 26, 2022).

[6] Kuroiwa Y. Interview with Yuji Kuroiwa: a novel approach to the ageing challenge. Bull World Health Organ. (2017) 95:736–7. doi: 10.2471/BLT.17.031117, PMID: 29147053 PMC5677615

[7] My Me-Byo Health Record (in Japanese) Home page on the internet (n.d.). Available at: https://www.pref.kanagawa.jp/docs/fz7/cnt/f532715/p991437.html (Accessed January 7, 2023).

[8] The Kanagawa me-Byo prospective cohort study (in Japanese) [home page on the internet] (n.d.). Available at: https://www.me-byo-cohort.jp (Accessed December 31, 2022).

[9] Janz NK Becker MH. The health belief model: a decade later. Health Educ Q. (1984) 11:1–47. doi: 10.1177/109019818401100101, PMID: 6392204

[10] Araujo de Carvalho I Epping-Jordan J Pot AM Kelley E Toro N Thiyagarajan JA. Organizing integrated healthcare services to meet older people’s needs. Bull World Health Organ. (2017) 95:756–63. doi: 10.2471/blt.16.187617 29147056 PMC5677611

[11] Saito Y Nakamura S Tanaka A Watanabe R Narimatsu H Chung UI. Checking the validity and reliability of the Japanese version of the mini-cog using a smartphone application. BMC Res Notes. (2022) 15:222. doi: 10.1186/s13104-022-06101-4, PMID: 35752807 PMC9233764

[12] Borson S Scanlan JM Chen P Ganguli M. The mini-cog as a screen for dementia: validation in a population-based sample. J Am Geriatr Soc. (2003) 51:1451–4. doi: 10.1046/j.1532-5415.2003.51465.x, PMID: 14511167

[13] Borson S Scanlan J Brush M Vitaliano P Dokmak A. The mini-cog: a cognitive ‘vital signs’ measure for dementia screening in multi-lingual elderly. Int J Geriatr Psychiatry. (2000) 15:1021–7. doi: 10.1002/1099-1166(200011)15:11<1021::aid-gps234>3.0.co;2-6, PMID: 11113982

[14] Kobayashi T Morimoto T Shimanoe C Ono R Otani K Mawatari M. Development of a simple screening tool based on the 5-question geriatric locomotive function scale for locomotive syndrome. J Orthop Sci. (2022) 27:913–20. doi: 10.1016/j.jos.2021.05.001, PMID: 34090778

[15] Maruyama T Ekuni D Higuchi M Takayama E Tokuno S Morita M. Relationship between psychological stress determined by voice analysis and periodontal status: a cohort study. Int J Environ Res Public Health. (2022) 19:9489. doi: 10.3390/ijerph19159489, PMID: 35954845 PMC9368672

[16] Higuchi M Nakamura M Shinohara S Omiya Y Takano T Mitsuyoshi S. Effectiveness of a voice-based mental health evaluation system for mobile devices: prospective study. JMIR Form Res. (2020) 4:e16455. doi: 10.2196/16455, PMID: 32554367 PMC7399964

[17] Miyashita H Nakamura M Svensson AK Nakamura M Tokuno S Chung UI. Association between electroencephalogram-derived sleep measures and the change of emotional status analyzed using voice patterns: observational pilot study. JMIR Form Res. (2020) 4:e16880. doi: 10.2196/16880, PMID: 32515745 PMC7312246

[18] Manolio TA Collins FS Cox NJ Goldstein DB Hindorff LA Hunter DJ. Finding the missing heritability of complex diseases. Nature. (2009) 461:747–53. doi: 10.1038/nature08494, PMID: 19812666 PMC2831613

[19] Manolio TA Bailey-Wilson JE Collins FS. Genes, environment and the value of prospective cohort studies. Nat Rev Genet. (2006) 7:812–20. doi: 10.1038/nrg1919 16983377

[20] Takeuchi K Naito M Kawai S Tsukamoto M Kadomatsu Y Kubo Y. Study profile of the Japan multi-institutional collaborative cohort (J-Micc) study. J Epidemiol. (2020) 31:660–8. doi: 10.2188/jea.JE20200147, PMID: 32963210 PMC8593573

[21] Sawaguchi E Nakamura S Watanabe K Tsuno K Ikegami H Shinmura N. Covid-19-related stigma and its relationship with mental wellbeing: a cross-sectional analysis of a cohort study in Japan. Front Public Health. (2022) 10:1010720. doi: 10.3389/fpubh.2022.1010720, PMID: 36249227 PMC9558281

[22] Katayama K Narimatsu H. Prediction of female breast cancer incidence among the aging society in Kanagawa, Japan. PLoS One. (2016) 11:e0159913. doi: 10.1371/journal.pone.0159913, PMID: 27532126 PMC4988816

[23] Narimatsu H Sakaguchi M Nakamura S Katayama K. Future patient incidence in hemato-oncology: a study using data from cancer registries in Japan. Risk Manag Healthc Policy. (2020) 13:2407–14. doi: 10.2147/RMHP.S277207, PMID: 33173364 PMC7648558

[24] Beard JR Jotheeswaran AT Cesari M Araujo de Carvalho I. The structure and predictive value of intrinsic capacity in a longitudinal study of ageing. BMJ Open. (2019) 9:e026119. doi: 10.1136/bmjopen-2018-026119 PMC6830681 31678933

[25] Furukawa TA Kawakami N Saitoh M Ono Y Nakane Y Nakamura Y. The performance of the Japanese version of the K6 and K10 in the world mental health survey Japan. Int J Methods Psychiatr Res. (2008) 17:152–8. doi: 10.1002/mpr.257, PMID: 18763695 PMC6878390

[26] Ikeda S Shiroiwa T Igarashi A Noto S Fukuda T Saito S. Developing a Japanese version of the Eq-5d-5l value set. J Natl Inst Public Health. (2015) 64:47–55.

[27] Eq-5d-5l User Guide [Home Page on the Internet] (n.d.). Available at: https://euroqol.org/publications/user-guides/. (Accessed December 31, 2022).

[28] Ota A Li Y Yatsuya H Tanno K Sakata K Yamagishi K. Working cancer survivors’ physical and mental characteristics compared to cancer-free workers in Japan: a nationwide general population-based study. J Cancer Surviv. (2021) 15:912–21. doi: 10.1007/s11764-020-00984-7 33433855 PMC8519890

[29] Saito Y Nakamura S Tanaka A Watanabe R Narimatsu H Chung UI. Evaluation of the validity and reliability of the 10-meter walk test using a smartphone application among Japanese older adults. Front Sports Act Living. (2022) 4:904924. doi: 10.3389/fspor.2022.904924, PMID: 36267485 PMC9576938

